# Combined Effects of Heat and Cd^2+^ Stress on Growth, Physiology, and Transcriptomic Responses in *Sipunculus nudus*

**DOI:** 10.3390/ani16131991

**Published:** 2026-06-27

**Authors:** Jianqiang Huang, Ruzhou Zhong, Shaowen Yang, Chuangye Yang, Qingheng Wang, Yuewen Deng

**Affiliations:** 1 College of Fisheries, Guangdong Ocean University, Zhanjiang 524088, China; huangjianqiang@catas.cn (J.H.); 19820016939@163.com (R.Z.); yangsw@gdou.edu.cn (S.Y.); yangcy@gdou.edu.cn (C.Y.); dengyw@gdou.edu.cn (Y.D.); 2 Zhanjiang Experimental Station, Chinese Academy of Tropical Agricultural Sciences, Zhanjiang 524013, China; 3 Guangdong Provincial Key Laboratory of Aquatic Animal Disease Control and Healthy Culture, Zhanjiang 524088, China

**Keywords:** *Sipunculus nudus*, heat stress, Cd^2+^ exposure, bioaccumulation, transcriptomic responses

## Abstract

Heat and Cd^2+^ stress are major environmental challenges for marine benthic invertebrates. This study examined their combined effects on growth, physiology, and transcriptomic responses in the peanut worm (*Sipunculus nudus*). After 30 days, Cd^2+^ reduced survival at 26 °C without significantly affecting growth, whereas at 32 °C, both survival and growth declined with increasing Cd^2+^ concentration, indicating that heat stress exacerbates Cd^2+^ toxicity. Cd accumulation increased with exposure concentration but was not affected by temperature. Heat stress increased immune (AKP) and antioxidant (SOD, CAT) enzyme activities, although significant increases in SOD and CAT were observed only under Cd^2+^ exposure. AKP activity rose at low Cd^2+^ concentrations and fell at high Cd^2+^ concentrations at 26 °C, whereas no significant difference occurred at 32 °C between 0 and 0.25 mg/L Cd^2+^. At the same temperature, SOD and CAT activities were significantly higher under high Cd^2+^ exposure than under low Cd^2+^ exposure. Transcriptome analysis showed that Cd^2+^ exposure activated longevity-related pathways, protein processing, and translation initiation. Heat stress activated Jak-STAT signaling and endoplasmic reticulum protein processing while inhibiting the ribosome pathway. Under combined stress, pathways related to xenobiotic metabolism, nutrient digestion and absorption, and amino acid derivative metabolism were broadly suppressed. These results highlight that heat stress exacerbates Cd^2+^ toxicity, affecting growth, enzyme activity, and transcriptomic responses, and provide insights into the adaptive strategies of marine benthic organisms under the combined pressures of climate change and heavy metal pollution.

## Simple Summary

Heat stress and heavy metal pollution are important environmental challenges for marine benthic organisms. This study investigated how elevated temperature and cadmium (Cd^2+^) exposure, alone and in combination, affect the peanut worm (*Sipunculus nudus*). The results show that heat stress intensified the toxic effects of Cd^2+^, leading to reduced growth and survival. In addition, heat and Cd^2+^ altered immune and antioxidant enzyme activities and affected multiple metabolic and detoxification pathways at the transcriptomic level. Under combined stress, pathways related to xenobiotic metabolism, nutrient digestion and absorption, and amino acid metabolism were significantly suppressed. These findings suggest that warming conditions may increase the sensitivity of marine benthic organisms to heavy metal pollution, posing potential risks to their health and to aquaculture sustainability.

## 1. Introduction

*Sipunculus nudus* is an important marine fishery resource with high nutritional and medicinal value, with an annual production exceeding 20,000 tons in southern China [1], and it is often referred to as “marine cordyceps” [2]. It is widely distributed along the southern coast of China [3], including the Leizhou Peninsula and the Beibu Gulf. As a typical burrowing benthic invertebrate, *S. nudus* feeds primarily on organic matter in surface sediments [1]. This feeding strategy increases its susceptibility to accumulating environmental pollutants, particularly heavy metals, compared with pelagic organisms. Therefore, in addition to its economic importance, *S. nudus* is considered a potential bioindicator of marine sediment quality [4].

In recent years, seawater warming driven by global climate change and heavy metal contamination in coastal areas have emerged as major environmental stressors threatening marine ecosystems [5]. Temperature is a key factor regulating feeding, growth, and survival in marine invertebrates [6]. In the Leizhou Peninsula, a major aquaculture region for *S. nudus*, seawater temperatures have been reported to range from 17.23 to 33.23 °C [7], indicating that local populations may experience substantial thermal fluctuations under natural conditions. While moderate warming can enhance metabolic activity and growth [8], temperatures exceeding physiological thresholds can lead to reduced feeding, growth inhibition, and increased mortality [9,10]. Meanwhile, heavy metals are persistent and toxic pollutants that accumulate in ecosystems and exert long-term adverse effects on organisms [11].

Among various heavy metals, cadmium (Cd) has attracted considerable attention due to its high toxicity and strong bioaccumulation potential. Previous studies have reported widespread Cd contamination in major aquaculture areas of *S. nudus*. In the coastal waters of the Leizhou Peninsula, Cd concentrations in surface sediments have shown an increasing trend under anthropogenic influence [12], with ecological risk levels reaching high-risk categories in some regions [13]. Similarly, Cd contamination in the Beibu Gulf exhibits localized ecological risks resulting from both natural processes and human inputs [14]. Once entering organisms, Cd can induce oxidative stress by promoting the excessive production of reactive oxygen species (ROS), thereby damaging cellular components, and ultimately disrupting cellular structure and function [15]. For example, Cd exposure significantly increases ROS levels and oxidative damage indicators in clams (*Meretrix* spp.) and induces apoptosis [16]. In addition, Cd can disrupt energy metabolism, resulting in growth inhibition and reduced survival [17]. This pattern has also been observed in sea cucumbers (*Apostichopus japonicus*) exposed to elevated Cd levels [18]. Notably, temperature variation may further influence the bioavailability, uptake, and excretion of heavy metals, thereby modulating their accumulation and toxicity [19]. Consequently, seawater warming and heavy metal pollution may interact synergistically, imposing more severe combined stress on marine invertebrates.

Previous studies in marine invertebrates have shown that elevated temperature and heavy metal exposure can induce oxidative stress, physiological dysfunction, and growth inhibition [16,18]. However, although the effects of temperature [20] or single heavy metals [21] on *S. nudus* have been reported, information on the molecular responses to combined heat stress and Cd^2+^ exposure remains limited, and their underlying regulatory mechanisms are still poorly understood. In the present study, experimental temperatures were selected based on previously reported optimal growth performance of *S. nudus*, with 26 °C representing the ambient temperature and 32 °C simulating high-temperature stress [20]. Cd^2+^ concentrations (0, 0.25, and 3 mg/L) were chosen to cover a low to high sublethal range, with the low concentration representing environmentally relevant exposure levels reported in contaminated coastal environments [12] and the high concentration based on preliminary toxicity tests as a sublethal exposure level. Therefore, this study aimed to systematically evaluate the effects of these temperatures and Cd^2+^ concentrations on growth, survival, Cd accumulation, and immune and antioxidant responses in *S. nudus*, and transcriptome analysis was performed to elucidate the underlying molecular response mechanisms. This study provides a theoretical basis for understanding the adaptive strategies of marine benthic organisms under the combined pressures of climate change and heavy metal pollution.

## 2. Materials and Methods

### 2.1. Experimental Animals and Design

*S. nudus* individuals were collected from the intertidal zone of Haijiao Village, Leizhou City, Zhanjiang, Guangdong Province, China. The collection site is located in the Leizhou Peninsula, an important aquaculture area for *S. nudus*, where seawater temperatures have been reported to range from 17.23 to 33.23 °C and Cd contamination has been detected in coastal sediments [7,12]. Healthy individuals with intact body surfaces, active burrowing behavior, and uniform size were selected. All individuals were acclimated in filtered seawater for 3 days to allow gut clearance, thereby eliminating ingested sediment and ensuring accurate body weight measurement. The initial body weight was 1.65 ± 0.14 g (mean ± SD). The experiment was conducted in 20 L glass aquaria containing a 10 cm sand layer, with seawater salinity maintained at 35‰. Continuous aeration was provided, and water temperature was maintained at ±0.5 °C using thermostatic devices [22]. Each of the 18 aquaria contained 300 individuals, resulting in a total of 5400 individuals.

A two-factor completely randomized experimental design was applied, including two temperatures (26 °C and 32 °C) and three Cd^2+^ concentrations (0, 0.25, and 3 mg/L). Based on preliminary acute toxicity tests conducted at 32 °C, the 7-day safe concentration and median lethal concentration (LC_50_) of Cd^2+^ for *S. nudus* were estimated to be 0.246 mg/L and 4.910 mg/L, respectively. These preliminary tests were conducted prior to the main experiment to determine appropriate exposure levels for experimental design purposes. Accordingly, 0.25 mg/L and 3 mg/L were selected as the low and high Cd^2+^ exposure concentrations in the present study. The highest Cd^2+^ concentration was set at 3 mg/L to minimize mortality during the 30-day exposure period. After acclimation at 26 °C, individuals were randomly assigned to six treatment groups (three replicates each) to examine the effects of Cd^2+^, heat stress, and their interaction: control at 26 °C without Cd^2+^ (T26-C0); low and high Cd^2+^ at 26 °C (T26-C0.25, T26-C3); elevated temperature at 32 °C without Cd^2+^ (T32-C0); and combined Cd^2+^ and elevated temperature at 32 °C (T32-C0.25, T32-C3). Each replicate consisted of a separate tank, and individuals within a tank were considered the experimental unit. CdCl_2_·2.5H_2_O was used to prepare Cd^2+^ solutions. During the experiment, individuals were fed a formulated diet daily at 1% of total biomass [23]. Freshwater was added as needed to maintain salinity; Cd^2+^ concentrations were monitored and adjusted when necessary. Seawater was completely renewed weekly using freshly prepared seawater spiked with Cd^2+^ at the corresponding nominal concentrations for each treatment group to maintain stable exposure conditions throughout the experiment. After 30 days, survival was determined by counting the remaining live individuals. They were then transferred to clean seawater under the corresponding conditions for 3 days for gut clearance prior to body weight measurement. Body wall tissues were subsequently sampled, flash-frozen in liquid nitrogen, and stored at −80 °C for further analysis.

### 2.2. Growth Performance

At the end of the experiment, individuals were transferred to clean seawater without sediment for 3 days to allow gut clearance, thereby eliminating residual sediment and ensuring accurate body weight measurements. Survival was recorded for each treatment. Final body weight was measured for surviving individuals only. Survival rate (SR) and weight gain ratio (WGR) were calculated as follows:

*SR* (%) = 100 × *N_t_*/*N*_0_

*WGR* (%) = 100 × (*m_t_*–*m*_0_)/*m*_0_

where *N*_0_ and *N_t_* represent the initial and final numbers of individuals, respectively, and *m*_0_ and *m_t_* represent the initial and final body weights (g), respectively.

### 2.3. Determination of Cd^2+^ Concentration

Approximately 2 g of body wall tissue (defined in this study as the outer body wall excluding gut contents, including the epidermis and underlying muscle layers) was collected from each individual. Samples were digested using a microwave digestion system, and Cd^2+^ concentrations were determined according to the Chinese national standard GB 5009.268-2016 [24] (Method I). For sediment analysis, approximately 100 g of sediment samples were dried at 80 °C to constant weight, followed by complete digestion using a mixture of hydrochloric acid, nitric acid, hydrofluoric acid, and perchloric acid. Cd^2+^ concentration was determined according to GB/T 17141-1997 [25]. The bioconcentration factor (BCF) was calculated as follows:

*BCF* = *c_i_*/*c_e_*

where *c_i_* represents the Cd^2+^ concentration in the organism, and *c_e_* represents the Cd^2+^ concentration in the environment.

### 2.4. Enzyme Activity Assays

Crude enzyme extracts from body wall tissues were prepared according to previously described methods [20]. The activities of alkaline phosphatase (AKP), acid phosphatase (ACP), catalase (CAT), and superoxide dismutase (SOD) were determined using commercial assay kits following the manufacturer’s instructions. All kits were obtained from Nanjing Jiancheng Bioengineering Institute (Nanjing, China).

### 2.5. RNA Extraction, Library Construction, and Sequencing

Total RNA was extracted from body wall tissues using TRIzol reagent (Invitrogen, Carlsbad, CA, USA) following previously described methods [26]. RNA quality, integrity, and concentration were assessed using a Fragment Analyzer. Poly(A) mRNA was enriched using oligo(dT)-attached magnetic beads and subsequently fragmented. The resulting fragments were used as templates for first- and second-strand cDNA synthesis using random hexamer primers. After end repair and A-tailing, sequencing adapters were ligated to the cDNA fragments. The products were PCR-amplified, purified using AMPure XP beads, and dissolved in EB buffer. The purified double-stranded cDNA was denatured into single-stranded DNA and circularized to construct single-stranded circular DNA (ssDNA) libraries. Sequencing was performed on an Illumina HiSeq 2000 platform by BGI Genomics Co., Ltd. (Shenzhen, China).

### 2.6. Sequencing Data Processing and Analysis

Raw sequencing data were processed using SOAPnuke v1.5.2 [27] to remove reads containing adapters, reads with more than 10% unknown bases (N), and low-quality reads (defined as reads in which more than 50% of bases had a quality score < 15). The resulting clean reads were retained in FASTQ format. Clean reads were aligned to the reference genome using HISAT2 v2.0.4 [28]. Subsequently, Bowtie2 v2.2.5 [29] was used to map reads to the reference gene sequences, and gene and transcript expression levels were quantified using RSEM v1.2.12 [30]. Differentially expressed genes (DEGs) were identified using DESeq2 v1.4.5 [31], with thresholds of false discovery rate (FDR) < 0.05 and |log_2_ fold change| > 1. Gene Ontology (GO) (http://www.geneontology.org/, accessed on 8 June 2026) and Kyoto Encyclopedia of Genes and Genomes (KEGG) (https://www.kegg.jp/, accessed on 8 June 2026) pathway enrichment analyses were performed for the DEGs using the hypergeometric test implemented in the phyper function of R. GO terms and KEGG pathways with FDR < 0.05 were considered significantly enriched.

### 2.7. Statistical Analysis of Experimental Data

Experimental data, including growth performance, enzyme activities, and Cd^2+^ concentrations, were analyzed using IBM SPSS Statistics 27 after being organized and processed in Microsoft Excel 2021. Prior to statistical analysis, data were tested for normality using the Shapiro–Wilk test and for homogeneity of variance using Levene’s test. Differences among treatments were assessed using one-way analysis of variance (ANOVA). Additionally, a two-way ANOVA was performed to evaluate the main effects of temperature and Cd^2+^ concentration as well as their interaction on growth, survival, and other measured parameters. Under the same temperature condition, Duncan’s multiple range test was applied to compare Cd^2+^ treatments. Under the same Cd^2+^ concentration, differences between temperature treatments were evaluated using an independent-samples *t*-test. Differences were considered statistically significant at *p* < 0.05.

## 3. Results

### 3.1. Effects of Temperature and Cd^2+^ on Growth and Survival of S. nudus

**Figure 1:**
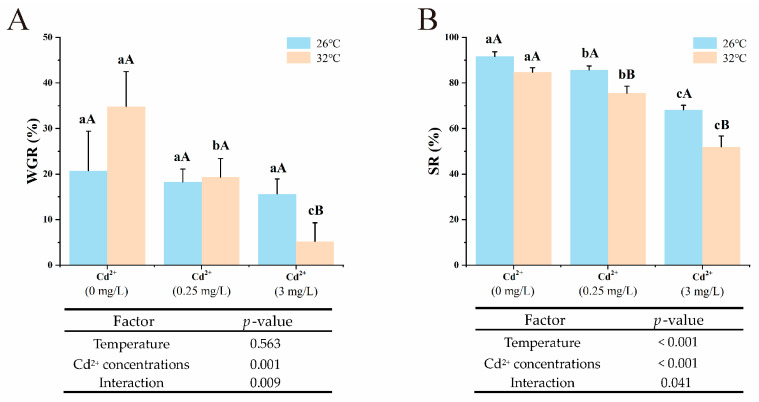
Effects of temperature and Cd^2+^ on the growth and survival of *S. nudus*. (**A**) WGR; (**B**) SR. Note: Different lowercase letters indicate significant differences among Cd^2+^ concentration groups at the same temperature (*p* < 0.05). Different uppercase letters indicate significant differences between temperature groups at the same Cd^2+^ concentration (*p* < 0.05). WGR: weight gain rate; SR: survival rate.

The weight gain ratio (WGR) of *S. nudus* under different treatments is shown in Figure 1A. At 26 °C, no significant differences in WGR were observed among Cd^2+^ concentrations (*p* > 0.05). In contrast, at 32 °C, WGR decreased significantly with increasing Cd^2+^ concentration (*p* < 0.05). At the same Cd^2+^ concentration, no significant differences in WGR were detected between 26 °C and 32 °C at 0 and 0.25 mg/L (*p* > 0.05), although the values were slightly higher at 32 °C. However, at 3 mg/L Cd^2+^, WGR at 32 °C was significantly lower than that at 26 °C (*p* < 0.05). Two-way ANOVA revealed a significant effect of Cd^2+^ concentration (*p* = 0.001) and a significant temperature × Cd^2+^ interaction (*p* = 0.009) on WGR, whereas the main effect of temperature was not significant (*p* = 0.563).

The survival rate (SR) of *S. nudus* is presented in Figure 1B. At both temperatures, SR decreased significantly with increasing Cd^2+^ concentration (*p* < 0.05), showing a clear dose–response relationship. At the same Cd^2+^ concentration, no significant difference in SR was observed between temperature groups in the control group (0 mg/L Cd^2+^). However, at 0.25 and 3 mg/L Cd^2+^, SR at 32 °C was significantly lower than that at 26 °C (*p* < 0.05). Two-way ANOVA indicated significant effects of temperature (*p* < 0.001), Cd^2+^ concentration (*p* < 0.001), and their interaction (*p* = 0.041) on SR.

### 3.2. Effects of Temperature and Cd^2+^ on Cd^2+^ Accumulation in the Body Wall of S. nudus

The Cd^2+^ content and bioconcentration factor (BCF) in the body wall of *S. nudus* are shown in Table 1. To account for potential effects of water exchange and sediment adsorption, Cd^2+^ concentrations in sediment interstitial water after 30 days were measured as the actual exposure levels. Cd^2+^ concentrations increased with increasing spiked doses in both water and sediment interstitial water, with sediment values slightly higher than those in the overlying water, likely due to residual Cd^2+^ retained during water exchange. In the control and elevated temperature groups without Cd^2+^ addition, Cd^2+^ was undetectable in water and sediment interstitial water, whereas trace amounts were detected in body wall tissues, likely reflecting pre-existing background accumulation. At the same Cd^2+^ exposure level, temperature had no significant effect on Cd^2+^ accumulation in the body wall (*p* > 0.05). In contrast, under the same temperature condition, Cd^2+^ content increased significantly with increasing exposure concentration (*p* < 0.05), showing a clear dose-dependent pattern, while the BCF decreased with increasing Cd^2+^ concentration. Two-way ANOVA further confirmed that Cd^2+^ concentration significantly affected Cd^2+^ accumulation in the body wall (*p* < 0.001), whereas neither temperature (*p* = 0.790) nor the temperature × Cd^2+^ interaction (*p* = 0.966) had a significant effect.

### 3.3. Effects of Temperature and Cd^2+^ on Immune and Antioxidant Enzyme Activities in S. nudus

**Figure 2:**
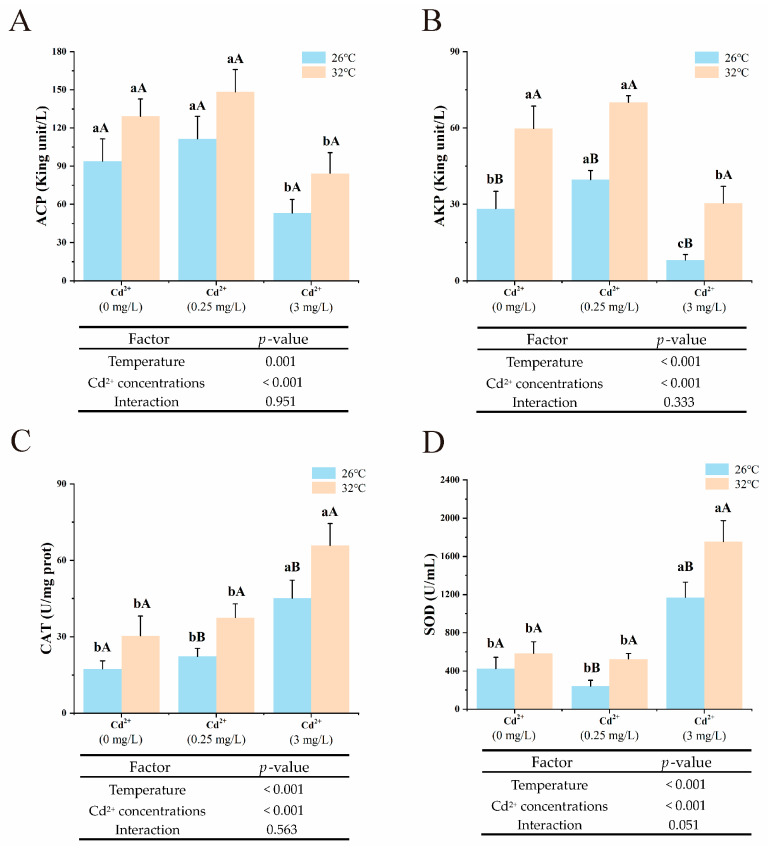
Effects of temperature and Cd^2+^ on enzyme activities in *S. nudus*. (**A**) ACP activity; (**B**) AKP activity; (**C**) CAT activity; (**D**) SOD activity. Note: Different lowercase letters indicate significant differences among Cd^2+^ concentration groups at the same temperature (*p* < 0.05). Different uppercase letters indicate significant differences between temperature groups at the same Cd^2+^ concentration (*p* < 0.05). ACP: acid phosphatase; AKP: alkaline phosphatase; CAT: catalase; SOD: superoxidase dimutase.

The activities of ACP, AKP, SOD, and CAT in the body wall tissues of *S. nudus* under different treatments are shown in Figure 2. At the same Cd^2+^ concentration, the activities of all four enzymes were higher at 32 °C than at 26 °C. Specifically, AKP showed significant differences at all Cd^2+^ concentrations (*p* < 0.05), whereas significant increases in CAT and SOD activities (*p* < 0.05) were observed only under Cd^2+^ exposure (0.25 and 3 mg/L). ACP showed no significant differences at any concentration (*p* > 0.05).

Under the same temperature condition, the responses of the four enzymes to Cd^2+^ exposure differed between functional groups. For ACP, activities at low Cd^2+^ concentrations (0 and 0.25 mg/L) were significantly higher than those at 3 mg/L (*p* < 0.05). For AKP at 26 °C, activity increased at low Cd^2+^ concentrations and decreased at the high concentration, with the highest value observed at 0.25 mg/L, whereas at 32 °C, AKP exhibited a pattern similar to that of ACP. In contrast, the antioxidant enzymes SOD and CAT showed a clear dose–response pattern, with activities at low Cd^2+^ concentrations (0 and 0.25 mg/L) being significantly lower than those at 3 mg/L (*p* < 0.05).

Two-way ANOVA showed significant effects of temperature and Cd^2+^ concentration on ACP, AKP, CAT, and SOD activities (*p* < 0.05). No significant temperature × Cd^2+^ interactions were observed for any of the four enzymes (ACP: *p* = 0.951; AKP: *p* = 0.333; CAT: *p* = 0.563; SOD: *p* = 0.051).

### 3.4. Transcriptome Sequencing Quality and Differential Expression Analysis

A total of 12 transcriptome libraries were successfully constructed from the body wall tissues of *S. nudus*, including the control group (T26-C0, 26 °C without Cd^2+^), the Cd^2+^ stress group (T26-C3, 26 °C with 3 mg/L Cd^2+^), the heat stress group (T32-C0, 32 °C without Cd^2+^), and the combined stress group (T32-C3, 32 °C with 3 mg/L Cd^2+^). Sequencing quality assessment showed that the Q20 values ranged from 96.37% to 98.31%, while the Q30 values ranged from 90.97% to 94.89% (Table 2). These results indicate high sequencing quality and confirm that the transcriptome data were suitable for subsequent analyses.

**Figure 3:**
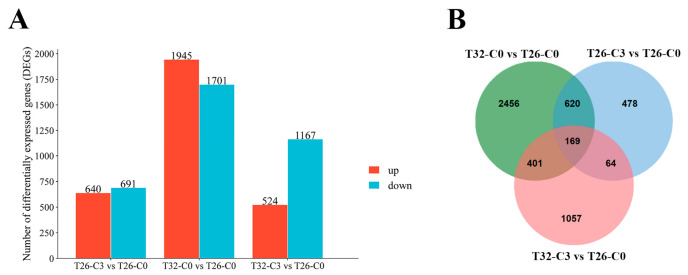
DEGs in *S. nudus* under Cd^2+^, heat stress, and combined stress. (**A**) Number of DEGs identified in each treatment compared with the control group (T26-C0, 26 °C without Cd^2+^). T26-C3: Cd^2+^ stress group (26 °C with 3 mg/L Cd^2+^); T32-C0: heat stress group (32 °C without Cd^2+^); T32-C3: combined stress group (32 °C with 3 mg/L Cd^2+^). “Up” and “Down” indicate the numbers of upregulated and downregulated genes, respectively. (**B**) Venn diagram showing the overlap of DEGs among the three treatment groups.

Based on the high-quality sequencing data, differential expression analysis was performed among treatments. The number of DEGs identified in each comparison is shown in Figure 3A. Compared with the control group, 1331 DEGs were identified in the Cd^2+^ stress group, including 640 upregulated and 691 downregulated genes. The heat stress group showed 3646 DEGs, including 1945 upregulated and 1701 downregulated genes, whereas the combined stress group showed 1691 DEGs, including 524 upregulated and 1167 downregulated genes.

Venn diagram analysis (Figure 3B) revealed that 169 DEGs were shared among the Cd^2+^ stress group, heat stress group, and combined stress group. Pairwise comparisons identified 233 shared DEGs between the Cd^2+^ stress group and the combined stress group, 789 between the heat stress group and the Cd^2+^ stress group, and 570 between the heat stress group and the combined stress group. In addition, 2456, 478, and 1057 unique DEGs were detected in the heat stress group, Cd^2+^ stress group, and combined stress group, respectively. These results indicate that *S. nudus* exhibits distinct transcriptional responses to Cd^2+^ stress, heat stress, and their combined effects. The large numbers of treatment-specific DEGs further suggest that each stress condition induced a unique transcriptional regulatory pattern. Notably, although the combined stress group exhibited fewer DEGs than the heat stress group, it still contained 1057 uniquely expressed DEGs, indicating that the transcriptional response to combined stress was not simply a subset of, or additive to, the responses induced by the individual stressors.

### 3.5. Transcriptomic Responses of S. nudus to Cd^2+^ Stress

**Figure 4:**
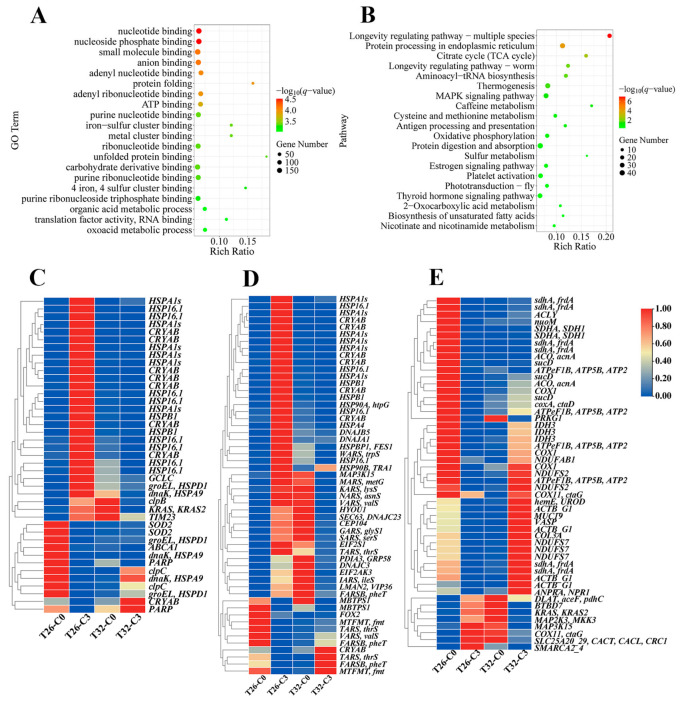
Transcriptomic responses of *S. nudus* to Cd^2+^ stress. (**A**) GO enrichment bubble plot of DEGs. (**B**) KEGG pathway enrichment bubble plot of DEGs. (**C**) Expression patterns of genes in longevity-related pathways. (**D**) Expression patterns of genes in protein processing-related pathways. (**E**) Expression patterns of genes in energy metabolism-related pathways. T26-C0: control group (26 °C without Cd^2+^); T26-C3: Cd^2+^ stress group (26 °C with 3 mg/L Cd^2+^); T32-C0: heat stress group (32 °C without Cd^2+^); T32-C3: combined stress group (32 °C with 3 mg/L Cd^2+^).

GO enrichment analysis of DEGs between the Cd^2+^ stress group and the control group showed significant enrichment in functions related to nucleotide and ATP binding, metal ion and iron–sulfur cluster binding, protein folding and binding, and organic acid and oxoacid metabolic processes (Figure 4A). In addition, translation factor activity and RNA binding were also enriched.

KEGG pathway analysis indicated that DEGs were significantly enriched in longevity-regulating pathways across multiple species, longevity-regulating pathways in worms, protein processing in the endoplasmic reticulum, the citrate cycle (TCA cycle), aminoacyl-tRNA biosynthesis, thermogenesis, and the MAPK signaling pathway (Figure 4B). Gene expression pattern analysis further showed that, in the Cd^2+^ stress group, 70.73% of genes in longevity-related pathways were upregulated, including *HSPA1s*, *HSP16.1*, *ClpB*, *CRYAB*, and *GCLC*, which was higher than the upregulation proportions in the control group (26.83%), the heat stress group (12.20%), and the combined stress group (9.76%) (Figure 4C). In pathways associated with protein processing and translation initiation (e.g., protein processing in the endoplasmic reticulum and aminoacyl-tRNA biosynthesis), 79.63% of genes were upregulated in the Cd^2+^ stress group, including *HSPA1s*, *CRYAB*, *DNAJB5*, and *DNAJA1*, which exceeded the upregulation proportions in the control group (18.52%), the heat stress group (35.19%), and the combined stress group (9.26%) (Figure 4D). In contrast, 82.35% of genes involved in energy metabolism pathways (e.g., the TCA cycle and thermogenesis) were downregulated in the Cd^2+^ stress group, including *ATP5B*, *ATP2*, *SDHA*, and *ACTB_G1*. The downregulation proportion was higher than those in the control group (19.61%) and the combined stress group (39.22%), and comparable to that in the heat stress group (84.31%) (Figure 4E).

### 3.6. Transcriptomic Responses of S. nudus to Heat Stress

**Figure 5:**
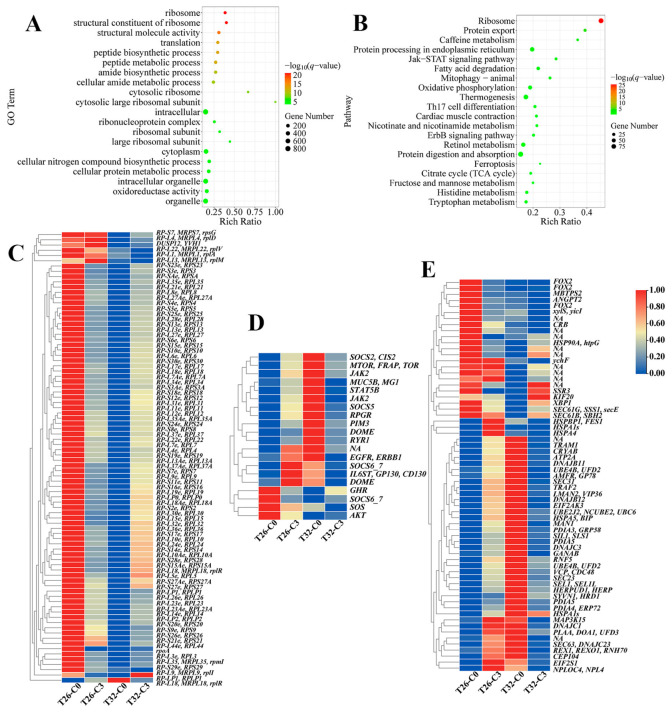
Transcriptomic responses of *S. nudus* to heat stress. (**A**) GO enrichment bubble plot of DEGs. (**B**) KEGG pathway enrichment bubble plot of DEGs. (**C**) Expression patterns of genes in ribosome pathways. (**D**) Expression patterns of genes in immune response regulation pathways. (**E**) Expression patterns of genes in endoplasmic reticulum protein processing pathways. T26-C0: control group (26 °C without Cd^2+^); T26-C3: Cd^2+^ stress group (26 °C with 3 mg/L Cd^2+^); T32-C0: heat stress group (32 °C without Cd^2+^); T32-C3: combined stress group (32 °C with 3 mg/L Cd^2+^).

GO enrichment analysis of DEGs between the heat stress group and the control group showed significant enrichment in ribosome-related functions, including ribosome, structural constituent of ribosome, and ribosomal subunit. DEGs were also enriched in structural molecule activity, translation, peptide and amide biosynthetic and metabolic processes, as well as intracellular organelle-related components (Figure 5A). In addition, oxidoreductase activity, cellular nitrogen compound biosynthetic processes, and cellular protein metabolic processes were also significantly enriched.

KEGG pathway analysis revealed that DEGs were significantly enriched in ribosome, protein export, protein processing in the endoplasmic reticulum, the Jak-STAT signaling pathway, and fatty acid degradation (Figure 5B). Gene expression pattern analysis further showed that, in the heat stress group, 80.00% of genes in the Jak-STAT signaling pathway were upregulated, including *SOCS*, *MTOR*, *JAK2*, and *STAT*, which was higher than the upregulation proportions in the control group (20.00%), the Cd^2+^ stress group (30.00%), and the combined stress group (5.00%) (Figure 5D). In the protein processing in the endoplasmic reticulum pathway, 60.00% of genes were upregulated in the heat stress group, including *TRAM1*, *HSP*, and *DNAJC*, which exceeded the upregulation proportions in the control group (35.38%), the Cd^2+^ stress group (26.15%), and the combined stress group (10.77%) (Figure 5E). In contrast, 98.84% of genes in the ribosome pathway, mainly encoding ribosomal protein subunits, were downregulated in the heat stress group, which was higher than the downregulation proportions in the control group (1.16%), the Cd^2+^ stress group (90.70%), and the combined stress group (83.72%) (Figure 5C).

### 3.7. Transcriptomic Responses of S. nudus to Combined Stress

**Figure 6:**
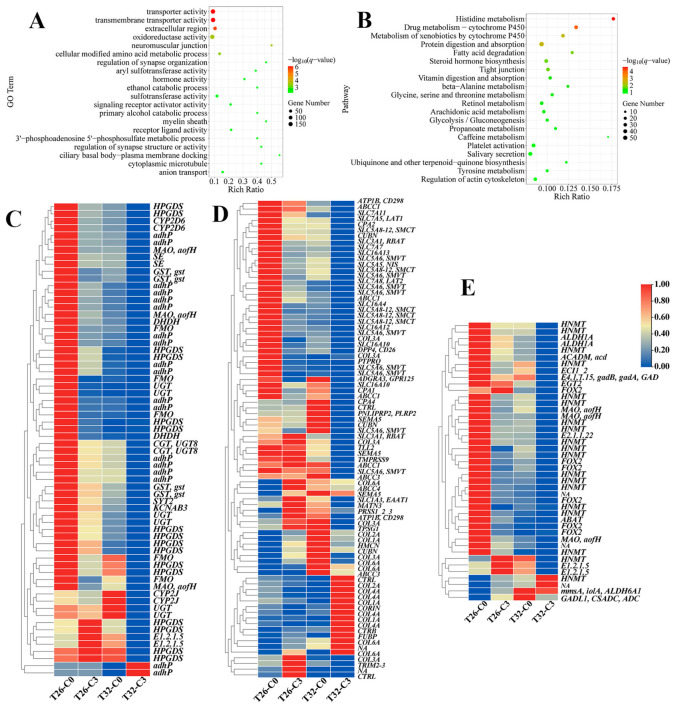
Transcriptomic responses of *S. nudus* to combined stresses. (**A**) GO enrichment bubble plot of DEGs. (**B**) KEGG pathway enrichment bubble plot of DEGs. (**C**) Expression patterns of genes in xenobiotic detoxification-related pathways. (**D**) Expression patterns of genes in nutrient digestion and absorption-related pathways. (**E**) Expression patterns of genes in amino acid derivative metabolism-related pathways. T26-C0: control group (26 °C without Cd^2+^); T26-C3: Cd^2+^ stress group (26 °C with 3 mg/L Cd^2+^); T32-C0: heat stress group (32 °C without Cd^2+^); T32-C3: combined stress group (32 °C with 3 mg/L Cd^2+^).

GO enrichment analysis of DEGs between the combined stress group and the control group showed significant enrichment in functions related to transporter activity, transmembrane transporter activity, extracellular region, oxidoreductase activity, and sulfotransferase activity, as well as hormone activity, receptor ligand activity, and anion transport (Figure 6A). DEGs were also enriched in the cellular-modified amino acid metabolism, the ethanol catabolic process, and during the regulation of synapse structure or activity.

KEGG pathway analysis revealed that DEGs were significantly enriched in histidine metabolism, drug metabolism-cytochrome P450, metabolism of xenobiotics by cytochrome P450, protein digestion and absorption, and fatty acid degradation (Figure 6B). Additional enriched pathways included steroid hormone biosynthesis, tight junction, vitamin digestion and absorption, and β-alanine metabolism. Gene expression pattern analysis showed that, in the combined stress group, 96.97% of genes involved in xenobiotic metabolism pathways were downregulated, including *HPGDS*, *CYP2D6*, *adhP*, *GST*, and *UGT*, which was higher than the downregulation proportions in the control group (12.12%), the Cd^2+^ stress group (80.30%), and the heat stress group (83.33%) (Figure 6C). In nutrient digestion and absorption pathways, 80.95% of genes were downregulated in the combined stress group, including members of the ABCC and SLC transporter families, which exceeded the downregulation proportions in the control group (45.24%), the Cd^2+^ stress group (69.05%), and the heat stress group (65.48%) (Figure 6D). Similarly, 93.02% of genes in amino acid derivative metabolism pathways were downregulated in the combined stress group, including *HNMT*, *ALDH*, *FOX2*, and *MAO*, which was higher than the downregulation proportions in the control group (16.28%), the Cd^2+^ stress group (81.40%), and the heat stress group (81.40%) (Figure 6E).

## 4. Discussion

The present study demonstrates that both temperature and Cd^2+^ exposure significantly influence the growth and survival of *S. nudus*. At 26 °C, Cd^2+^ exposure did not affect weight gain but significantly reduced survival, indicating that within the optimal temperature range, Cd^2+^ primarily impacts organismal viability through toxicity-related effects [17]. Previous studies have shown that elevated temperature may enhance Cd uptake and reduce its excretion by regulating related genes, thereby increasing intracellular Cd accumulation and toxicity [32]. However, in the present study, temperature did not significantly affect Cd^2+^ accumulation in the body wall of *S. nudus*. This result suggests that the enhanced toxicity observed under combined stress was not necessarily associated with increased Cd accumulation. Nevertheless, at 32 °C, Cd^2+^ toxicity was markedly exacerbated, as reflected by further declines in survival and a significant reduction in weight gain with increasing Cd^2+^ concentration, indicating a significant interaction between temperature and Cd^2+^ exposure, as supported by the two-way ANOVA. The increased toxicity under combined stress may be attributed to multiple mechanisms. Elevated temperature increases metabolic activity and oxygen consumption, promoting reactive oxygen species (ROS) production and inducing oxidative stress [33]. Cd^2+^, as a non-essential metal, further enhances ROS generation and activates antioxidant defense responses. In the present study, SOD and CAT activities increased with increasing Cd^2+^ concentration, suggesting that *S. nudus* upregulated its antioxidant system to counteract Cd-induced oxidative stress. However, despite this compensatory response, excessive ROS production may still exceed the organism’s antioxidant capacity, leading to lipid peroxidation, protein damage, and mitochondrial dysfunction [34]. In addition, high temperature may impair detoxification processes by affecting the expression or activity of key molecules, such as metallothioneins (MTs), thereby reducing metal-binding and clearance capacity [35]. Moreover, immune cells of marine invertebrates are more sensitive to Cd^2+^ under elevated temperature, exhibiting increased mortality and functional impairment, which may contribute to the pronounced decline in survival under combined stress [36]. Under low or absent Cd^2+^ exposure, the slightly higher weight gain observed at 32 °C suggests that moderate warming alone can promote growth [20]. However, this effect was largely offset at high Cd^2+^ levels (3 mg/L), indicating that under severe stress, energy allocation shifts from growth to stress defense, particularly toward oxidative stress responses [18].

The results indicate that Cd^2+^ accumulation in the body wall of *S. nudus* is primarily governed by environmental Cd^2+^ concentration rather than temperature, exhibiting a clear dose-dependent pattern. The decline in the bioaccumulation factor with increasing Cd^2+^ concentration is consistent with patterns reported in other benthic invertebrates [37,38]. It is generally attributed to the saturation of metal uptake at high exposure levels, along with enhanced excretion and detoxification processes such as metallothionein binding and glutathione-mediated pathways [37]. Despite the potential influence of temperature on metabolic activity, no significant temperature effect on Cd^2+^ accumulation was observed under constant exposure conditions. Similar findings have been reported in the Pacific oyster, where temperature did not alter metal distribution among tissues [39], suggesting that metal accumulation remains relatively stable within this heat range. This stability may also be associated with the structural characteristics of the body wall, which is rich in mucus and polysaccharides that maintain metal-binding capacity under moderate temperature fluctuations [40]. Trace amounts of Cd^2+^ detected in the body wall of the control and warming-only groups are likely associated with pre-existing environmental accumulation prior to laboratory exposure. *S. nudus* was collected from coastal areas of the Leizhou Peninsula, where low-level Cd contamination has been reported in sediments [12,13], suggesting potential environmental exposure of local populations. Similar background Cd levels have also been reported in benthic invertebrates inhabiting coastal sediments [41]. Therefore, the low Cd^2+^ concentrations observed in these groups were considered to reflect background accumulation rather than experimental exposure.

Elevated temperature enhanced the activities of non-specific immune enzymes (ACP and AKP) and antioxidant enzymes (SOD and CAT) in *S. nudus*. At the same Cd^2+^ concentration, enzyme activities were consistently higher at 32 °C than at 26 °C, indicating that heat stress promotes immune and antioxidant responses, likely through increased metabolic rates and enzymatic activities [42]. In response to Cd^2+^ exposure, AKP exhibited a biphasic pattern, with stimulation at low concentrations (0.25 mg/L) and inhibition at higher levels. This response likely reflects the activation of immune defenses under mild oxidative stress, followed by enzyme inhibition and functional impairment when stress exceeds the physiological tolerance threshold [43]. In contrast, SOD and CAT activities increased progressively with Cd^2+^ concentration, indicating an enhanced antioxidant response to counteract ROS accumulation. These results are consistent with previous studies showing that benthic invertebrates maintain redox homeostasis under multiple environmental stressors through the upregulation of antioxidant enzymes [44], highlighting the central role of oxidative stress regulation in coping with combined temperature and heavy metal exposure.

Transcriptome analysis revealed substantial transcriptional remodeling in *S. nudus* under both single and combined stressors, with clear differences in gene expression patterns among treatments. Interestingly, the combined stress treatment triggered fewer DEGs than heat stress alone, suggesting that the transcriptional response to simultaneous temperature and Cd^2+^ exposure was not simply additive [45,46]. This pattern may reflect a more selective regulatory strategy, in which *S. nudus* prioritizes key stress-response pathways and essential physiological processes, rather than broadly activating all genes responsive to individual stressors [47]. Similar patterns have been reported in marine invertebrates exposed to multiple environmental stressors [48]. These findings highlight that combined stressors produce a unique transcriptional response that cannot be predicted from single stressor effects alone, emphasizing the complexity of molecular adaptation under multiple environmental challenges.

Under Cd^2+^ stress alone, DEGs were mainly enriched in nucleotide and ATP binding, metal ion and iron-sulfur cluster binding, protein folding, and organic acid metabolism. These results indicate that Cd^2+^ disrupts energy metabolism, metal homeostasis, and protein stability in *S. nudus*. This is consistent with a toxicity mechanism driven by oxidative stress and protein damage [49]. KEGG analysis further showed upregulation of longevity-regulating and endoplasmic reticulum protein-processing pathways, accompanied by downregulation of the TCA cycle and thermogenesis pathways. This pattern reflects a shift from energy production to stress defense, representing a typical energy reallocation strategy under heavy metal stress [50].

Heat stress primarily enriched pathways related to ribosome function, protein homeostasis, and redox regulation. Approximately 80% of genes in the Jak-STAT signaling pathway were upregulated, consistent with its role in immune and stress regulation [51]. This suggests that heat stress enhances cellular defense through activation of immune signaling and modulation of protein folding and processing [52]. At the same time, most ribosomal genes were downregulated, despite their enrichment at the pathway level, indicating suppression of basal protein translation activity. This likely conserves energy and prioritizes the synthesis of stress-related proteins under elevated temperature [53].

Under combined heat and Cd^2+^ stress, DEGs were mainly enriched in transporter activity, oxidoreductase activity, and catabolic processes. KEGG analysis revealed widespread downregulation of pathways involved in detoxification, nutrient digestion and absorption, and amino acid metabolism under combined stress, indicating a transcriptional response distinct from that observed under individual stressors. The downregulation of detoxification-related genes suggests that cellular detoxification processes may be affected under multifactorial stress conditions [32]. Notably, key detoxification genes such as *CYP450* and *GST* were suppressed, which may involve upstream regulatory pathways like *Nrf2*/*Keap1*, a major sensor of oxidative stress, although further studies are needed to confirm this [54,55]. Although transcriptomic changes indicate modulation of genes related to Cd uptake and excretion, no significant effect of temperature on Cd accumulation in the body wall was observed in our physiological measurements. This suggests that changes in gene expression do not necessarily translate into measurable differences in tissue Cd content over the experimental period, possibly due to compensatory mechanisms, tissue-specific distribution, or post-transcriptional regulation [56,57]. Likewise, the reduced expression of genes associated with nutrient transport and metabolism may reflect altered nutrient utilization and energy metabolism, which is consistent with the observed reductions in growth and survival [58]. In addition, the suppression of amino acid metabolism pathways may be associated with changes in cellular metabolic status and stress adaptation [59,60]. Together, these transcriptomic changes suggest that combined heat and Cd^2+^ stress induces broad alterations in metabolic and physiological regulatory processes in *S. nudus*.

## 5. Conclusions

Heat stress exacerbated Cd^2+^ toxicity in *S. nudus*, resulting in reduced growth and survival. Heat stress stimulated immune and antioxidant enzyme activities, while Cd^2+^ exhibited concentration-dependent effects. Transcriptome analysis showed that Cd^2+^ exposure activated longevity-related pathways, protein processing, and translation initiation; heat stress activated Jak-STAT signaling pathway and endoplasmic reticulum protein processing, but inhibited the ribosome pathway; and combined stress broadly suppressed xenobiotic metabolism, nutrient digestion and absorption, and amino acid derivative metabolism. These findings suggest that *S. nudus* responds to combined heat and Cd^2+^ stress through coordinated molecular regulation, highlighting the synergistic effects of heat stress on Cd^2+^ toxicity and its associated physiological disruption. Future studies should further investigate the physiological functions of key candidate genes and pathways identified in this study, as well as the long-term adaptive responses of *S. nudus* to multiple environmental stressors under changing climatic conditions.

## Figures and Tables

**Table 1:** The concentration of Cd^2+^ in the body wall of *S. nudus* and its living environment in each experimental group.

Group	Temperature/°C	Cd^2+^ in Water/(mg/L)	Cd^2+^ in Sediment/ (mg/L)	Cd^2+^ in Body Wall Tissue /(mg/kg)	Bioconcentration Factor
T26-C0	26	-	-	0.11 ± 0.03 ^cA^	-
T26-C0.25	26	0.25	0.40	46.30 ± 8.05 ^bA^	115.75
T26-C3	26	3.00	3.96	158.00 ± 21.22 ^aA^	39.90
T32-C0	32	-	-	0.17 ± 0.04 ^cA^	-
T32-C0.25	32	0.25	0.33	45.30 ± 3.40 ^bA^	137.27
T32-C3	32	3.00	4.12	155.00 ± 10.01 ^aA^	37.62
*p*-value of two-way ANOVA					
Temperature	-	-	-	0.790	-
Cd^2+^ concentrations	-	-	-	<0.001	-
Interaction	-	-	-	0.966	-

Note: “-” indicates no result. Different lowercase letters indicate significant differences among Cd^2+^ concentration groups at the same temperature (*p* < 0.05); different uppercase letters indicate significant differences between temperature groups at the same Cd^2+^ concentration (*p* < 0.05). T26-C0: 26 °C without Cd^2+^; T26-C0.25: 26 °C with 0.25 mg/L Cd^2+^; T26-C3: 26 °C with 3 mg/L Cd^2+^; T32-C0: 32 °C without Cd^2+^; T32-C0.25: 32 °C with 0.25 mg/L Cd^2+^; T32-C3: 32 °C with 3 mg/L Cd^2+^.

**Table 2:** Quality statistics of filtered reads.

Sample	Total Raw Read (M)	Total Clean Read (M)	Clean Read Q20 (%)	Clean Read Q30 (%)	Clean Read Ratio (%)
T26-C0-a	43.82	42.35	98.27	94.78	96.64
T26-C0-b	43.82	42.24	98.18	94.50	96.40
T26-C0-c	45.57	43.52	98.28	94.82	95.49
T26-C3-a	45.57	43.49	96.38	90.98	95.42
T26-C3-b	45.57	42.76	96.56	91.43	93.83
T26-C3-c	45.57	42.56	96.62	91.56	93.38
T32-C0-a	45.57	42.82	96.44	91.14	93.96
T32-C0-b	45.57	42.78	96.37	90.97	93.88
T32-C0-c	45.57	42.97	96.62	91.56	94.28
T32-C3-a	45.57	43.26	98.31	94.89	94.93
T32-C3-b	45.57	43.36	98.19	94.56	95.13
T32-C3-c	43.82	42.27	98.06	94.25	96.46

Note: “a, b, c” represent different biological replicates. T26-C0: 26 °C without Cd^2+^; T26-C3: 26 °C with 3 mg/L Cd^2+^; T32-C0: 32 °C without Cd^2+^; T32-C3: 32 °C with 3 mg/L Cd^2+^.

## Data Availability

The data that support the findings of this study are available on request from the corresponding author. The data are not publicly available due to privacy restrictions.
